# Thymoproteasome-Expressing Mesenchymal Stromal Cells Confer Protective Anti-Tumor Immunity *via* Cross-Priming of Endogenous Dendritic Cells

**DOI:** 10.3389/fimmu.2020.596303

**Published:** 2021-01-19

**Authors:** Jean-Pierre Bikorimana, Nehme El-Hachem, Abed El-Hakim El-Kadiry, Jamilah Abusarah, Natasha Salame, Riam Shammaa, Moutih Rafei

**Affiliations:** ^1^ Department of Microbiology, Infectious Diseases and Immunology, Université de Montréal, Montreal, QC, Canada; ^2^ Centre Hospitalier Universitaire (CHU) Ste-Justine Research Center, Université de Montréal, Montreal, QC, Canada; ^3^ Genomics Institute of Precision Medicine, American University of Beirut, Beirut, Lebanon; ^4^ Department of Pharmacology and Physiology, Université de Montréal, Montreal, QC, Canada; ^5^ Department of Microbiology and Immunology, McGill University, Montreal, QC, Canada; ^6^ Department of Biomedical Sciences, Université de Montréal, Montreal, QC, Canada; ^7^ Department of Family and Community Medicine, University of Toronto, Toronto, ON, Canada; ^8^ Canadian Centers for Regenerative Therapy, Toronto, ON, Canada; ^9^ Intellistem Technologies Inc., Toronto, ON, Canada; ^10^ Molecular Biology Program, Université de Montréal, Montreal, QC, Canada

**Keywords:** mesenchymal stromal cell, thymoproteasome, cancer vaccine, antigen cross-presentation, cross-priming, efferocytosis, clodronate

## Abstract

Proteasomes are complex macromolecular structures existing in various forms to regulate a myriad of cellular processes. Besides degrading unwanted or misfolded proteins (proteostasis), distinct immune functions were ascribed for the immunoproteasome and thymoproteasome (TPr) complexes. For instance, antigen degradation during ongoing immune responses mainly relies on immunoproteasome activity, whereas intrathymic CD8 T-cell development requires peptide generation by the TPr complex. Despite these substantial differences, the functional contribution of the TPr to peripheral T-cell immunity remains ill-defined. We herein explored whether the use of mesenchymal stromal cells (MSCs) engineered to exhibit altered proteasomal activity through *de novo* expression of the TPr complex can be exploited as a novel anti-cancer vaccine capable of triggering potent CD8 T-cell activation. Phenotypic and molecular characterization of MSC-TPr revealed a substantial decrease in MHCI (H2-K^b^ and H2-D^d^) expression along with elevated secretion of various chemokines (CCL2, CCL9, CXCL1, LIX, and CX3CL1). In parallel, transcriptomic analysis pinpointed the limited ability of MSC-TPr to present endogenous antigens, which is consistent with their low expression levels of the peptide-loading proteins TAP, CALR, and PDAI3. Nevertheless, MSC-TPr cross-presented peptides derived from captured soluble proteins. When tested for their protective capacity, MSC-TPr triggered modest anti-tumoral responses despite efficient generation of effector memory CD4 and CD8 T cells. In contrast, clodronate administration prior to vaccination dramatically enhanced the MSC-TPr-induced anti-tumoral immunity clearly highlighting a refractory role mediated by phagocytic cells. Thus, our data elute to a DC cross-priming-dependant pathway in mediating the therapeutic effect of MSC-TPr.

**Graphical Abstract:**
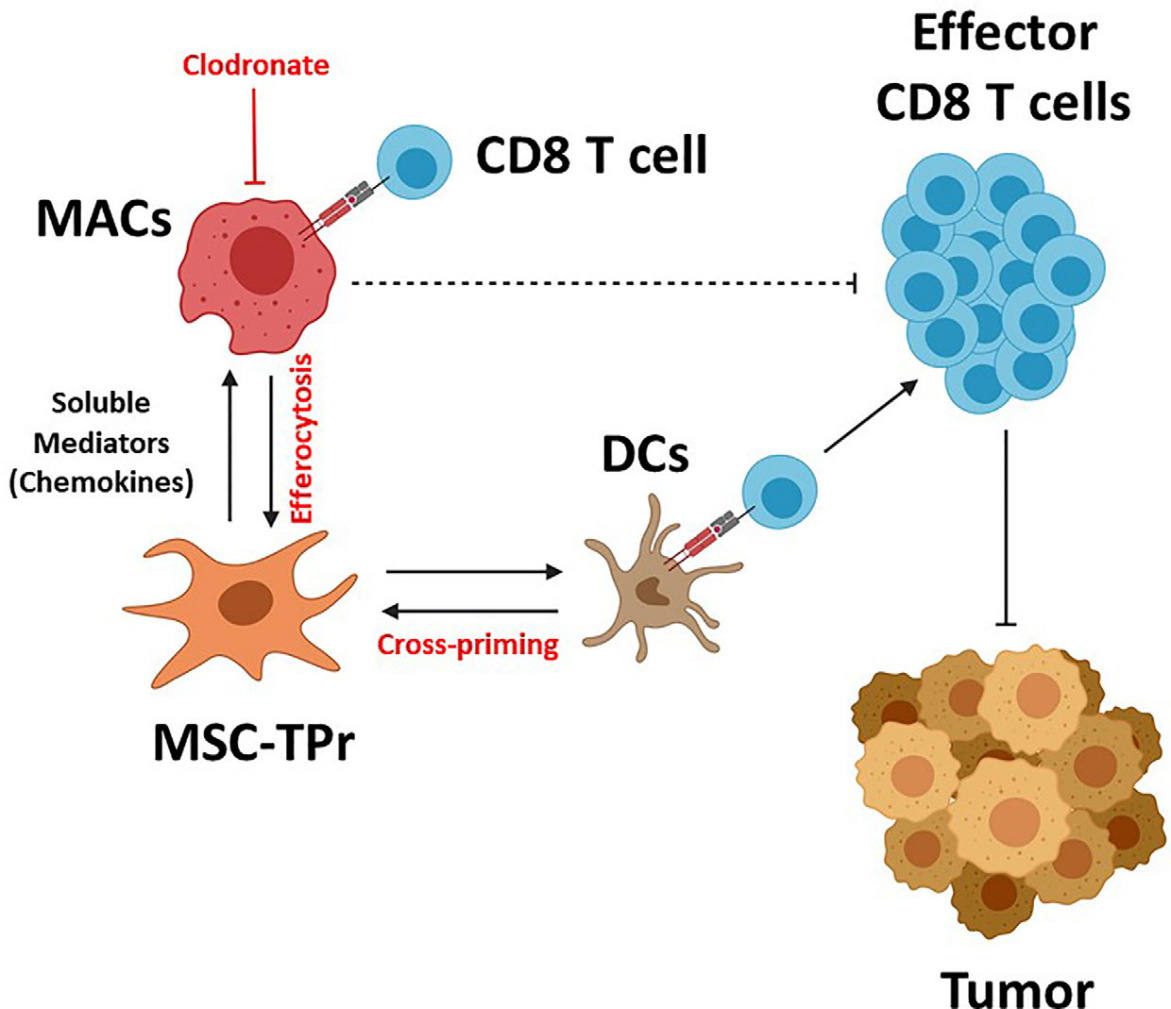
MSC-TPr administration to immunocompetent mice leads to the recruitment of myeloid cells (macrophages and DCs) *via* a myriad of soluble mediators including chemokines. Although DC recruitment and cross-priming is beneficial to trigger anti-tumoral immunity, the interaction between MSC-TPr and monocytes/macrophages impairs T-cell activation. Depletion of macrophages by clodronate re-establishes full anti-tumoral immunity leading to complete protection.

## Introduction

Initially, the proteasomal machinery was believed to strictly coordinate proteolysis *via* the degradation of aberrant cytoplasmic proteins (1). This perception quickly evolved with the emergence of additional proteasomal functions including the regulation of transcriptional activities, cell cycle and division, DNA repair, immunity, development/differentiation as well as organelle biogenesis (1–4). In contrast to yeast, which harbors a single proteasomal form, mammals can express three types of proteasomes: i) the constitutive proteasome (CPr: β1, β2, and β5), ii) the immunoproteasome (IPr: β1i, β2i, and β5i), and iii) the thymoproteasome (TPr: β1i, β2i, and β5t) (1, 5). Whereas the CPr complex is expressed in all eukaryotic cells, the IPr subunits are constitutively detected in antigen-presenting cells or assembled *de novo* in eukaryotic cells following interferon (IFN)-gamma stimulation. Compared to the CPr, the proteolytic activity of the IPr readily generates peptides fitting snugly in major histocompatibility (MHC)I grooves, which ensures efficient immune surveillance and pathogen clearance by CD8 T cells (1). TPr biogenesis, on the other hand, is restricted to cortical thymic epithelial cells (cTECs) (1, 6, 7). Its role is vital for positive selection of CD8 thymocytes as mice deficient in *β5t* exhibit aberrant CD8 T-cell development (CD4 T cells are not affected) despite normal thymic architecture and MHCI expression on cTECs (7, 8). The latter observations in *β5t*-deficient mice led to the conclusion that the unique and poor chymotrypsin-like activity of the TPr provides a set of MHCI-associated peptides completely distinct from those generated by the CPr or IPr complexes and dedicated to CD8 T-cell development (7, 8). It is however unclear whether the benefits of TPr are merely limited to the development of functional CD8 T cells or can also be exploited in immunotherapy to activate peripheral CD8 T cells. Additional studies are therefore required to investigate whether molding of peripheral CD8 T-cell immunity can be mediated by the TPr.

To this extent, we opted for culture-adapted non-hematopoietic mesenchymal stromal cells (MSCs) as a working model since they: i) can be harvested by a simple low volume aspirate from mice, ii) exhibit rapid *in vitro* proliferation, iii) require minimal culture conditions, iv) display minimal senescence through multiple passages, and v) are highly permissive to a variety of genetic engineering methods (9, 10). Besides, MSCs can switch gears and behave as conditional antigen-presenting cell upon IFN-gamma licensing, buttressing their rationale use in the context of antigen presentation (11). We show in this study that TPr-expressing MSCs (MSC-TPr) can indeed capture and cross-present peptides derived from soluble antigens. This acquired antigen presentation potential confers decent anti-tumor protection in immunocompetent mice. However, these elicited anti-tumor responses result from a delicate balance between phagocyte-mediated efferocytosis and endogenous DC cross-priming.

## Materials and Methods

### Animals and Ethics

All female C57BL/6NCrl and Balb/c mice (6–8 weeks old) were purchased from the Jackson Laboratory (Bar Harbor, ME, USA) and housed in a pathogen-free environment at the animal facility of the Institute for Research in Immunology and Cancer (IRIC). All experimental procedures and protocols were approved by the Animal Ethics Committee of Université de Montréal.

### Cell Lines and Reagents

The EG.7 T-cell lymphoma, the AP2 retroviral plasmid and the 293-GP2 packaging system were kindly provided by Dr. Jacques Galipeau (University of Wisconsin-Madison, WI, USA). The A20 B-cell lymphoma was purchased from ATCC (Manassas, VA). All cell culture media and reagents were purchased from Wisent Bioproducts (St-Jean-Baptiste, QC, Canada). All flow-cytometry antibodies were purchased from BD Biosciences (San Jose, CA, USA). These include PE anti-CD11b (clone KH95, catalog no. 553574), PE anti-CD11c (clone HL3; catalog no. 557401), PE anti-CD31 (clone MEC 13.3; catalog no. 553373), APC anti-CD44 (clone IM7; catalog no. 561862), PE anti-CD45 (clone 30-F11; catalog no. 553081), PerCP-Cy™5.5 anti-CD62L (clone MEL-14; catalog no. 560513), PE anti-CD73 (clone TY/23; catalog no. 550741), anti-CD90.1 (clone OX-7; catalog no. 551401), PE anti-CD105 (clone MJ7/18; catalog no. 562759), PE anti-H2-K^b^ (clone AF6-88.5; catalog no. 553570), PE anti-H2-D^b^ (clone KH95; catalog no. 553574), PE anti-I-A^b^ (clone AF6-120.1; catalog no. 553552), PE anti-CD80 (clone 16-10A1; catalog no. 553769), PE anti-CD86 (clone GL1; catalog no. 553692), and PE anti-PD-L1 (clone MIH5; catalog no. 558091). All ELISA kits and western blot antibodies were purchased from R&D Systems (Minneapolis, MN, USA). The 25-D1.16 antibody was purchased from Biolegend (San Diego, CA, USA). PolyFect and the RNeasy mini kit were purchased from Qiagen (Toronto, ON, Canada). The chicken egg white ovalbumin (OVA) protein, Cell Lytic™ lysis buffer, lipopolysaccharide (LPS) and Accutase^®^ were purchased from Sigma-Aldrich (St-Louis, MI, USA). Recombinant GM-CSF was purchased from PeproTech (Rocky Hill, NJ, USA). Polybrene was purchased from Cedarlane (Burlington, ON, Canada). The OVA Alexa Fluor™ 647 Conjugate was purchased from ThermoFisher (Mississauga, ON, Canada). The SIINFEKL peptide was synthesized by Genscript (Piscataway, NJPiscataway, NJ, USA). The chemokine array was purchased from RayBiotech (Peachtree Corners, GA, USA). The CD8 T-cell isolation kit was purchased from StemCell Technologies (Vancouver, BC, Canada). The Bradford reagent was purchased from Bio-Rad (Hercules, CA, USA). Liposomes and liposome-Clodronate were purchased from Liposoma Research (Amersterdam, The Netherlands).

### Generation of Bone Marrow–Derived Dendritic Cells

To generate dendritic cells (DCs), the tibia/femur bones of 8 weeks old C57BL/6 female mice were flushed. Collected bone marrow (BM) cells were plated in 10-cm Petri dishes containing 10 ml of complete RPMI 1640 medium (10% fetal bovine serum (FBS), 1% sodium pyruvate, 1% HEPES, 1% non-essential amino acids, 1% l-glutamine, 1% penicillin-streptomycin, and 5 μM β-mercaptoethanol) supplemented with 50 ng/ml of recombinant GM-CSF (PeproTech). The media was changed at days 3 and 6 of culture prior to the induction of DC maturation using 1 ng/ml of LPS (Sigma) added at day 8 for 12 h.

### Generation of BM-Derived MSCs

In order to generate MSCs, femurs of 6–8 weeks old female C57BL/6 mice were isolated and flushed with Alpha Modification of Eagle’s Medium (AMEM) supplemented with 10% FBS, and 50 U/ml of penicillin-streptomycin in a 10 cm cell culture dish (CellStar), then incubated at 37°C. Two days later, non-adherent cells were removed and the media was replaced every 3 to 4 days until the cells reached 80% confluency. Adherent cells were detached using 0.05% trypsin and expanded until a uniform MSC population is obtained. Generated MSCs were validated by flow-cytometry for the expression of CD31, CD44, CD45, CD73, CD90, and CD105.

### Engineering MSCs to Express the TPr Subunits

**Figure 1:**
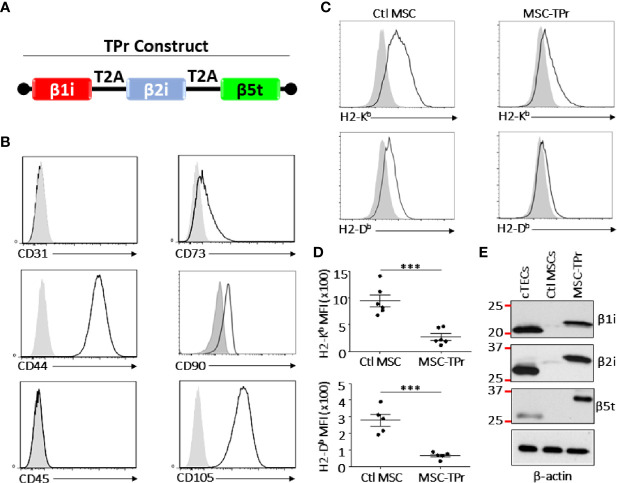
Engineering and characterizing MSC-TPr. **(A)** A cartoon representing the cloning strategy used to generate the TPr retroviral construct. **(B)** Phenotypic analysis of MSC-TPr after gene-modification. **(C, D)** Comparative analysis for the expression levels of MHCI molecules on Ctl MSC versus MSC-TPr. **(E)** Representative Western-blot depicting the expression of the three TPr subunits in 1) cTECs (positive control), 2) Ctl MSCs, and 3) MSC-IPr. For the experiment in **(D)**, n *=* 6/group with ***P < 0.001.

The AP2 retroviral construct, containing the green fluorescent protein (eGFP) as a marker for retroviral expression, was designed to contain the genes coding for the three murine TPr subunits (β1i, β2i, and β5t) separated by the viral T2A sequence (**Figure 1A**) (12). Both AP2 and pVSV-G (encoding the viral envelop protein) were used to co-transfect the GP2-293 packaging cell line using PolyFect (Qiagen) according to the manufacturer’s protocol. Supernatants containing the virus were collected 48 h post-transfection and centrifuged at 1,500 rpm for 5 min at 4°C to remove cell debris. An ultracentrifugation at 25,000 rpm for 90 min at 4°C was then conducted to concentrate the virus 10-fold. Passage 5 MSCs were plated at 50–60% and transduced with the concentrated virus in a minimal volume containing 5 μg/ml of Polybrene (Cedarlane). Transduction efficiency was confirmed by eGFP expression and immunoblotting of the TPr subunits. Similar steps were followed using the empty AP2 construct backbone to generate control (Ctl) MSCs.

### Cytokine and Chemokine Analyses

For cytokine and chemokine profiling, 15 cm culture petri dishes containing 80–90% confluent MSC-β5t or Ctl MSCs were grown in serum-free media for 24 h at 37°C. Collected supernatants were then analyzed using luminex by Eve Technologies (Calgary, AB, CA) or commercially-available chemokine arrays (RayBiotech) according to manufacturer’s instructions.

### RNA Extraction and Sequencing

Total RNA was isolated from 10^6^ cells using RNeasy^®^ mini kit (QIAGEN) according to manufacturer’s instructions. Following total RNA quantification by QuBit (ABI), 500 ng of total RNA was used for library preparation. Quality of total RNA was assessed with the BioAnalyzer Nano (Agilent) and all samples had a RIN above 8. Library preparation was done with the KAPA mRNAseq stranded kit (KAPA, Cat no. KK8420). Ligation was made with 9 nM final concentration of Illumina index and 10 PCR cycles was required to amplify cDNA libraries. Libraries were quantified by QuBit and BioAnalyzer. All librairies were diluted to 10 nM and normalized by qPCR using the KAPA library quantification kit (KAPA; Cat no. KK4973). Libraries were pooled to equimolar concentration. Sequencing was performed with the Illumina Hiseq2000 using the Hiseq Reageant Kit v3 (200 cycles, paired-end) using 1.7 nM of the pooled library. Around 40 M paired-end PF reads were generated per sample. Library preparation and sequencing was made at the IRIC Genomics Platform.

### Bioinformatics Analysis

Raw RNA-seq counts from reads aligned to the mouse genome (mm10 assembly) were generated with Htseq-count (PMID: 25260700). Differentially expressed genes between TPr and Ctl MSCs were calculated by DESeq2 (PMID: 25516281). Pre-ranked gene set enrichment was performed as recommended for RNA-seq data (PMID: 16199517). Custom R scripts were used to filter highly redundant biological processes. A false discovery rate of 0.05 was considered as an acceptable threshold for further investigation. All analyses were conducted in R (v3.6) or Python (v3.7) programming language. The ggplot2, ClusterProfiler and dplyr packages were used for data visualization. Student’s *t-test* was performed for normally distributed data to compute the *p*-value and the Benjamini-Hochberg procedure was used for adjusting the statistical inference of multiple comparisons.

### Detection of MHC-Peptide Complexes on the Surface of Pulsed MSC-TPr

To detect the SIINFEKL/MHC-I complex at the cell surface, MSC-TPr or Ctl MSCs were pulsed with the OVA protein (5 mg/ml) or the SIINFEKL peptide (2.5 μg/ml) at 37°C for different time points. The supernatants were discarded and the cells were washed twice with PBS prior to adding fresh medium. The signal for SIINFEKL/MHCI complex on cell surface was monitored by flow-cytometry at 1, 3, 6, 9, and 24 h post-pulsing using the 25-D1.16 antibody (Biolegend). To detect the increase in peptide-MHCI complex formation on H2-K^b^ and H2-D^d^ molecules, the cells were pulsed with OVA (5 mg/ml) for 1 h prior to detection of the H2-K^b^/H2-D^b^ levels by flow-cytometry at 1, 3, 6 and 24 h post-washing (chase).

### Monitoring Antigen Uptake and Processing

To evaluate the cells ability to up-take antigens, MSC-TPr or Ctl MSCs were seeded at 4 × 10^4^ cells/well in a 12 well plate (Corning) and incubated with 1 μg/ml of Alexa Fluor^®^647-conjugated OVA. At the end of incubation time, treated cells were then detached and washed four times with cold PBS containing 2% FBS. Fluorescence was detected by BD FACS Diva on CANTOII and analyzed by FlowJoV10.

### Antigen Presentation Assay

For the antigen cross-presentation assay *in vitro*, Ctl MSCs or MSC-TPr cells were seeded at 25 × 10^3^ cells per well in a 24-well plate (Corning). The following day, plated cells were washed and pulsed with 5 mg/ml of OVA or 2.5 μg/ml of the SIINFEKL peptide. At the end of the pulsing period, the cells were washed prior to their co-culture with 10^6^/ml CD8 T-cells purified from the spleen of OT-I mouse or immunized mice using the CD8α^+^ positive isolation kit according to the manufacturer’s protocol. The supernatants were collected three days later to quantify IFN-gamma levels by ELISA.

### Peritoneal Lavage and CellTrace™ Analysis

To specifically assess *in vivo* efferocytosis, 10^6^ CellTrace™-labelled MSC-TPr were *intraperitoneally (IP)-*injected in immunocompetent 6–8 weeks old female *C57BL/6 mice* (n *=* 3/group). Clodronate or control liposomes (50μl/20g mouse) were delivered the day before (13). Four hours following MSC admninistration, mice were sacrificed and peritoneal lavage was conducted using 30 ml of serum-free RPMI. Collected cells were centrifuged at 1 500 rpm for 10 min and the cell pellets washed twice with PBS. Recovered cells were then analyzed by flow-cytometry to detect CD11b^+^ cells exhibiting a positive CellTrace™ signal.

### Generation of Heat-Killed MSCs

Ctl MSCs and MSC-TPr were heat-killed as previously described (13). Briefly, ready for injection MSCs (re-suspended in sterile PBS) were incubated in a water bath at 50°C for 15 min. Cell death was confirmed by annexin-V and the absence of peptide-MHC complexes on their cell surface.

### Cancer Cell Lysate Preparation

To prepare the A20 cell lysate, 50 × 10^6^ cells were collected by centrifugation at 1 500 rpm for 10 min and the cell pellets washed twice with PBS to remove traces of FBS. The pellets underwent 5 cycles of freeze and thaw using liquid nitrogen and boiling water respectively. After removing large particles using 70 μM cell strainers followed by an additional filtration with a 0.45 μM filter, the lysate was quantified using Bradford reagent then aliquoted and stored at -80°C until use.

### Immunization and Tumor Challenge

For all prophylactic vaccination studies, 6–8 weeks old female C57BL/6 mice (n *=* 10/group) or Balb/c mice (n *=* 10/group) were IP-injected at day 0 and 14 with OVA- (5 mg/ml) or tumor lysate- (1 mg/ml) pulsed MSC-TPr versus Ctl MSCs. Two weeks following the second vaccination, mice were subcutaneously (SC) challenged with 5 × 10^5^ EG.7 or A20 cells and tumor growth was assessed two to three times per week. To detect memory CD4 and CD8 T cells, blood samples isolated from immunized mice were stained with CD44 and CD62L and analyzed by flow-cytometry.

To block efferocytosis during immunization, 6–8 weeks old female C57BL/6 mice (n *=* 10/group) were IP-injected with liposome-clodronate or control liposome one day prior to immunization (50μl/20g mouse) (13). Two weeks later, the same process was repeated, followed by tumor challenge (5 × 10^5^ EG.7 cells) one week following the second immunization. All tumor-free mice were then re-challenged 10 weeks later (total of two EG.7/A20 challenges). A similar approach was followed to block DC cross-priming except that animals were IP-injected with 30 μg of anti-CD11c antibodies or isotype control.

### Statistical Analysis

*P*-values were calculated using the one-way analysis of variance (ANOVA) for most experiments unless otherwise mentioned. Statistical significance is represented with asterisks: *P < 0.05, **P < 0.01, ***P < 0.001. Statistical tests used for bioinformatic analysis are described in their corresponding sections.

## Results

### Phenotypic Characterization of TPr-Expressing MSCs

**Figure 2:**
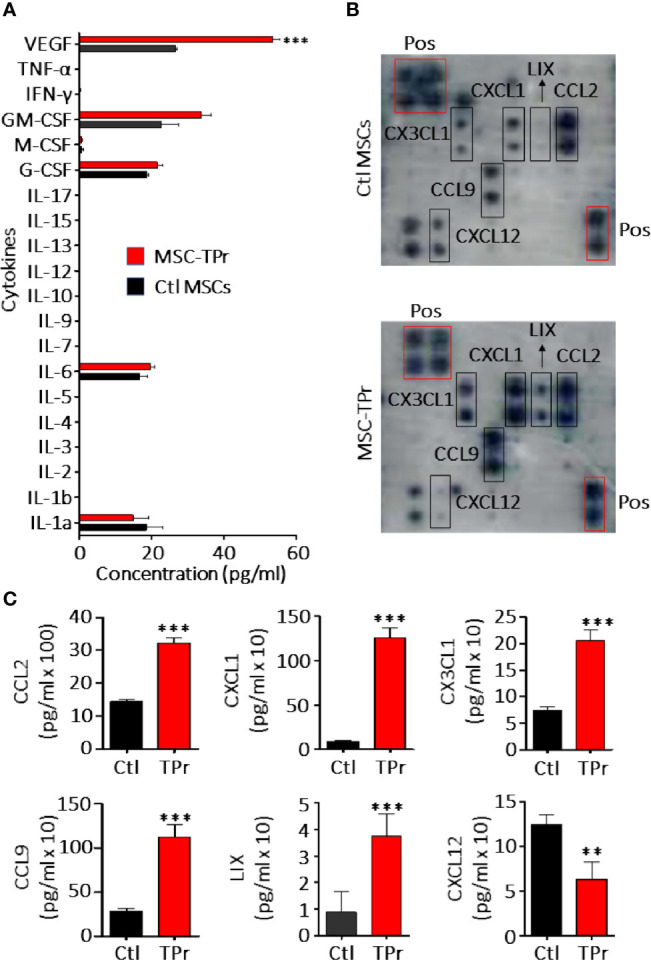
Analysis of MSC-TPr secretome. **(A)** Luminex quantification of various cytokines secreted by Ctl (black) or MSC-TPr (red). **(B)** Analysis of various chemokines secreted by Ctl versus TPr-expressing MSCs using commercial chemokine arrays. **(C)** Quantification of chemokines selected from **(B)**. For **(A, C)**, n = 6/group with **P < 0.01 and ***P < 0.001.

Although genetic modification of MSCs to express the TPr subunits (β1i, β2i, and β5t; **Figure 1A**) did not alter their original mesenchymal phenotype (**Figure 1B**; **Figure S1**), lower levels of H2-K^b^ and H2-D^b^ were detected on the surface of MSC-TPr in comparison to Ctl MSCs (**Figures 1C, D**). In addition, expression of the TPr by MSCs (**Figure 1E**) did not induce MHCII (I-A^b^), CD80, CD86 or PD-L1 expression (**Figure S2**), nor caused unusual alterations in their responsiveness to IFN-gamma treatment (**Figure S3**). When analyzed for their secretome profile, MSC-TPr expressed higher levels of VEGF (**Figure 2A**), in addition to CCL2, CCL9, CXCL1, LIX, and CX3CL1 while CXCL12 was decreased (**Figures 2B, C**). These results indicate that TPr expression in MSCs alters their MHCI expression and secretion of various soluble immune mediators.

### TPr Expression May Limit the Ability of MSCs to Process and Present Endogenous Antigens

**Figure 3:**
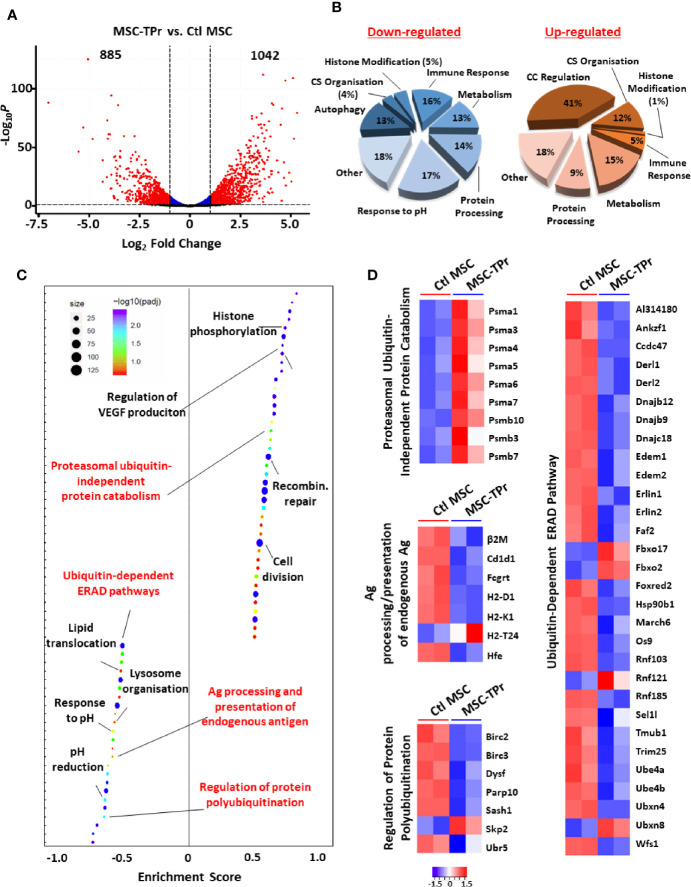
Transcriptomic analysis of MSC-TPr. **(A)** Volcano plot showing the estimated fold changes (x-axis) versus the minus log10 of the adjusted *p*-values (y-axis) from DEseq analysis. Significant genes with absolute value of log2 fold-changes greater or equal to 0.5 are shown in red. **(B)** Major biological processes groups modulated in MSC-TPr compared to Ctl MSCs. CS refers to cytoskeleton whereas CC means cell cycle. **(C)** Plot showing the enriched GO biological processes from an unbiased GSEA analysis of the differentially expressed genes between MSC-TPr and Ctl MSCs. The FDR threshold is set to 0.05. Features are ranked by the enrichment score from the KS test (x-axis). **(D)** A series of heat-maps representing the z-scored expression level of the differentially expressed genes from various processes. Up-regulated and down-regulated genes are highlighted in red and blue respectively.

Given the pleiotropic functions mediated by the proteasome, we next questioned the impact of *de novo* TPr expression on the overall transcriptional profile of MSCs. In sum, 885 and 1042 genes were significantly down- and up-regulated respectively (**Figure 3A**) affecting various biological processes (**Figure 3B**) and pathways as shown by pre-ranked Gene Set Enrichment Analysis (**Figure 3C**). Given our interest in antigen presentation, we mainly focused on pathways related to protein degradation (**Figure 3D**) and observed enhanced expression of genes encoding for the alpha and beta subunits of the 20S proteasomal complex (*Psma1, Psma3-7*, *Psmb3*, *Psmb7*, and *Psmb10*). Interestingly, most of these genes are associated with the proteasome ubiquitin-independent protein catabolism pathway, which was recently reported to depend on β5t expression (14). This may suggest that in MSC-TPr, degradation of various client proteins may preferentially occur *via* the ubiquitin-independent pathway especially with the observed decrease in the expression of several genes regulating protein poly-ubiquitination (*Birc2*, *Birc3*, *Papr10*, and *Ubr5*; **Figure 3D**). These observations are also in agreement with the decreased ability of the MSC-TPr in processing and presenting endogenous antigens and the low expression levels of various genes involved in the endoplasmic reticulum-associated protein degradation (ERAD) pathway (**Figure 3D**). The relevance and/or significance for the existence of such ubiquitin-independent degradation pathway in MSC-TPr is unclear. However, this catabolic pathway may be a default mechanism for the degradation of various endogenous proteins involved in DNA and chromatin structure, metabolism, immunity, cell cycle and apoptosis, which may not require a fast-adaptive response for their proteostasis control (15). Overall, transcriptomic analysis suggests that TPr expression negatively impacts endogenous protein catabolism and presentation by MSCs, which explains the previously observed decrease in MHCI on their cell surface (**Figures 1C, D**).

### MSC-TPr Can Present Peptides Derived From Exogenous Soluble Proteins

**Figure 4:**
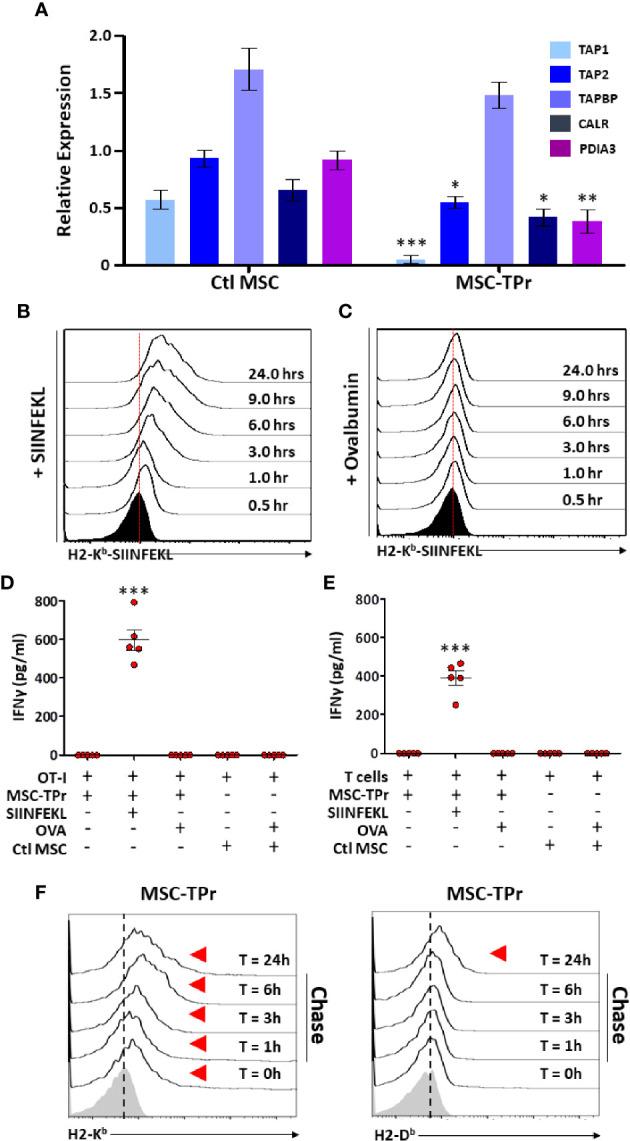
Evaluating the antigen cross-presentation ability of MSC-TPr. **(A)** Transcript quantification of genes involved in antigen presentation. **(B, C)** Representative flow-cytometry analysis of the SIINFEKL/H2-K^b^ complex following pulsing with the SIINFELK peptide **(B)** or OVA protein **(C)**. Black histograms represent the population in question without peptide/protein pulsing (t=0). The red dotted lines represent the threshold level according to non-pulsed controls. **(D, E)** An antigen-presentation assay conducted using MSC-TPr following pulsing with the SIINFEKL peptide (2.5 μg/ml) or OVA protein (5 mg/ml). Quantification of IFN-gamma production was conducted following MSC-TPr co-culturing with OT-I CD8 T cells **(D)** or CD8 T cells isolated from mice immunized using OVA-pulsed MSC-TPr. **(F)** Flow-cytometry analysis of H2-K^b^ and H2-D^b^ following OVA pulsing. For **(A, D, E)**, n = 5/group with *P < 0.05, **P < 0.01 and ***P < 0.001.

The intriguing observation of diminished endogenous antigen processing and presentation led us to quantify the expression of various genes involved in the peptide-loading machinery (16). With the exception of tapasin (*Tapbp*), expression of the transporter associated with antigen processing (*Tap*)*1* and *Tap2*, Protein-disulfide Isomerase-associated (*Pdai*)*3* and calreticulin (*Calr*) were down-regulated in MSC-TPr (**Figure 4A**). We next monitored the kinetics of SIINFEKL-H2-K^b^ complex formation at the cell surface of MSC-TPr following SIINFEKL or OVA pulsing. Although SIINFEKL pulsing increased the formation of the peptide-MHCI complex on the surface of MSC-TPr in a timely fashion (**Figure 4B**), the same SIINFEKL-MHC complex was undetected following OVA pulsing (**Figure 4C**) despite efficient protein uptake (**Figure S4A**). These observations are consistent with the impaired OT-I T-cell activation using OVA-pulsed MSC-TPr (**Figure 4D**). Since SIINFEKL is a strong and stable peptide immunogen unlikely to be generated by the poor chymotrypsin-like activity of the TPr complex, we hypothesized that CD8 T cells isolated from animals immunized using OVA-pulsed MSC-TPr would respond to other OVA-derived peptides without avail (**Figure 4E**). Given these unexpected data, we next validated the ability of MSC-TPr to present other OVA-derived peptides by assessing their cell surface level of MHCI molecules following protein pulsing (reflecting formation of MHC-peptide complexes). A pulse-chase experiment revealed the progressive appearance of H2-K^b^/H2-D^b^ levels on the surface of OVA-pulsed MSC-TPr (**Figure 4F**), which was substantially higher than Ctl MSCs (**Figure S4B**). Collectively, these results suggest that TPr expression in MSCs instills a distinct peptide repertoire unlikely to trigger peripheral CD8 T-cell activation.

### Prophylactic Vaccination of Macrophage-Depleted Animals Confers Potent Long-Term Protection Against T-Cell Lymphoma

**Figure 5:**
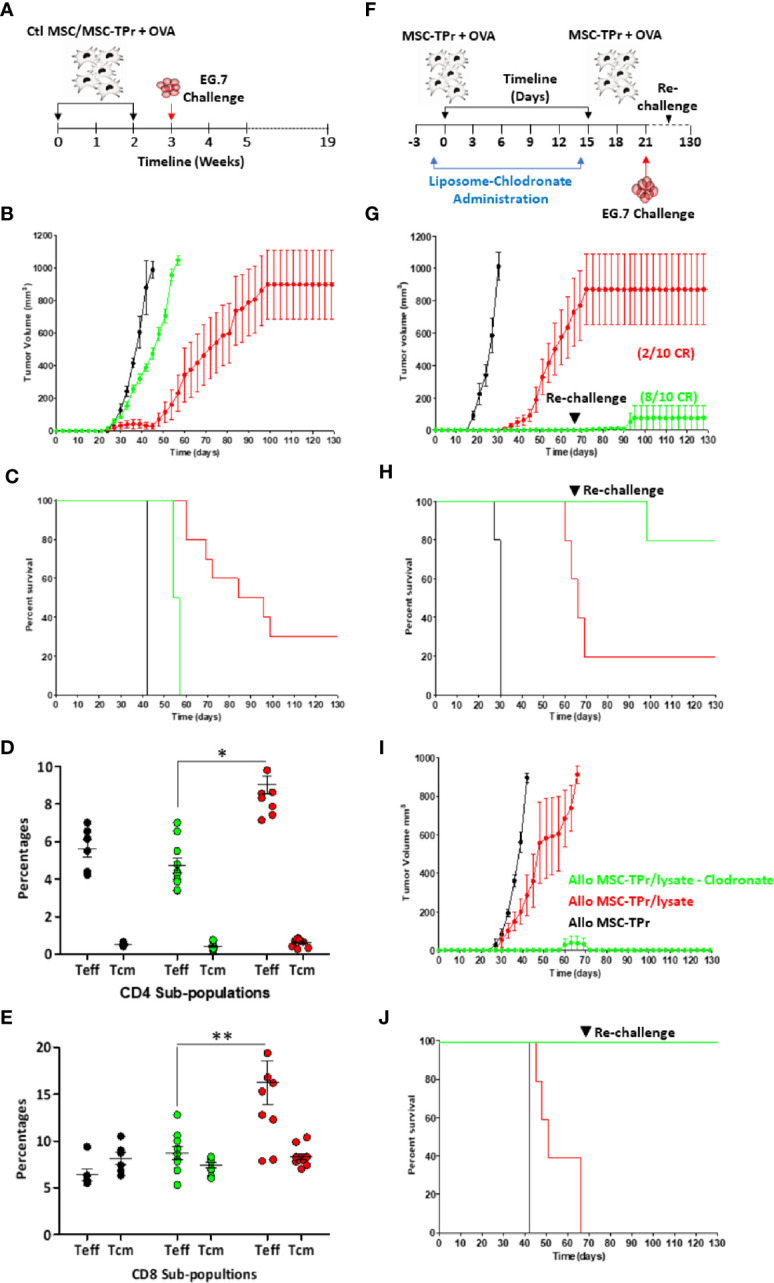
Testing the protective efficacy of MSC-TPr against T-cell lymphoma. **(A)** Schematic diagram representing the prophylactic vaccination schedule. **(B)** Prophylactic vaccination against EG.7 cells. C57BL/6 mice were vaccinated using OVA-pulsed Ctl MSCs (green) or MSC-TPr (red). Non-immunized animals injected with the EG.7 tumor cells are shown in black. **(C)** Kaplan-Meier survival curve of the experiment shown in **(B)**. **(D, E)** Flow-cytometry assessment of effector and central memory CD4 **(D)** and CD8 **(E)** T cells. Ctl mice are shown in black, Ctl MSCs in green and MSC-TPr in red. The main bars represent the mean ± SEM. **(F)** Schematic diagram representing clodronate use with prophylactic vaccination. **(G)** Prophylactic vaccination against EG.7 cells. Vaccinated C57BL/6 mice receiving liposome are shown in red whereas clodronate-treated mice are shown in green. Non-immunized control animals injected with the EG.7 tumor cells are shown in black. **(H)** Kaplan-Meier survival curve of the experiment shown in **(G)**. **(I)** Prophylactic vaccination against A20 cells. Vaccinated Balb/c mice receiving liposome are shown in red whereas clodronate-treated mice are shown in green. Non-immunized Ctl animals injected with the A20 tumor cells are shown in black. **(H)** Kaplan-Meier survival curve of the experiment shown in **(I)**. For **(B. C, G, J)**, n *=* 10/group. For **(D, E)** n *=* 7/group with *P < 0.05 and **P < 0.01.

Since MSC-TPr can efficiently uptake and process soluble OVA, yet are unable to activate CD8 T cells *in vitro*, we next interrogated their therapeutic utility against the E.G7 (OVA-expressing EL4 thymoma) T-cell lymphoma model (**Figure 5A**). In contrast to OVA-pulsed Ctl MSCs, which failed in providing a meaningful anti-tumor effect, prophylactic vaccination using OVA-pulsed MSC-TPr significantly delayed tumor growth with a 30% survival rate reached beyond 120 days (**Figures 5B, C**). To define the basis of this prolonged survival, we next quantified the levels of effector (CD44^hi^CD62L^-^) and central memory (CD44^hi^CD62L^+^) T cells in treated animals. The level of effector memory CD4 and CD8 T cells in the blood of mice vaccinated using OVA-pulsed MSC-TPr was 9 and 16%, respectively, in comparison with 4.8 and 9.2% in mice vaccinated using OVA-pulsed Ctl MSCs and 6 and 5.6% in un-vaccinated mice (**Figures 5D, E**).

The fact that MSC-TPr can elicit modest anti-tumor responses without any apparent ability to activate CD8 T cells *in vitro* strongly indicates cross-priming of “bystander” immune cells *in vivo*. Although the immunomodulatory function of MSCs is mostly driven by soluble mediators and/or contact factors (13, 17–19), previous pre-clinical studies revealed the importance of macrophages in supporting MSC-mediated immunosuppression either through efferocytosis of apoptotic MSCs or *via* IL-10 secretion in response to MSCs-derived chemokine interactome (13, 20). Considering the pivotal role of macrophages in promoting MSCs immunosuppression, we next examined the impact of MSC-TPr vaccination following monocyte/macrophage depletion by clodronate administration (**Figure 5F**). Indeed, phagocyte depletion enhanced the anti-tumor response induced by OVA-pulsed MSC-TPr in both syngeneic (**Figures 5G, H**) and allogeneic models (**Figures 5I, J**) despite secondary re-challenges. These data thus confirm a major suppressive role mediated by phagocytic cells in response to MSC-TPr vaccination.

### DC Cross-Priming Is Indispensable to the Anti-Tumor Effect of MSC-TPr

**Figure 6:**
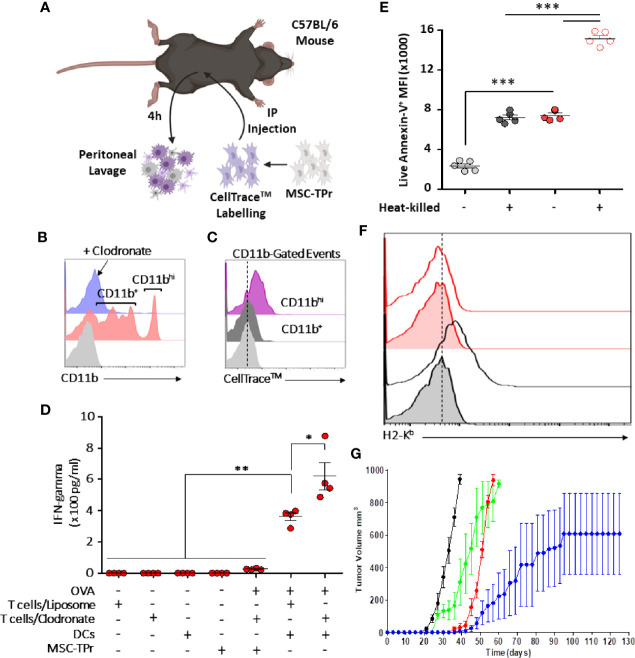
Evaluating the cross-priming ability of MSC-TPr. **(A)** Schematic diagram representing the experimental design for efferocytosis analysis. **(B)** Analysis of CD11b^+^ cells collected from the peritoneum of vaccinated mice. **(C)** Flow-cytometry assessment of CD11b^+^ exhibiting a CellTrace™ signal. **(D)** An antigen presentation assay conducted using DCs and CD8 T cells isolated from liposome-/clodronate-treated and vaccinated mice. **(E)** Quantification of the Annexin-V signal on live versus heat-killed Ctl (gray) or MSC-TPr (red). **(F)** Flow-cytometry assessment of H2-K^b^ on the surface of live (gray) versus heat-killed (red) MSC-TPr. **(G)** Prophylactic vaccination against EG.7 cells. C57BL/6 mice (n *=* 6/group) were vaccinated using OVA-pulsed heat-killed MSC-TPr (green) or following administration of CD11c to deplete DCs (red). Ctl MSCs are shown in black and OVA-pulsed MSC-TPr in blue. For **(D, E)**, n *=* 5/group with *P < 0.05, **P < 0.01 and ***P < 0.001.

Since clodronate administration enhances dramatically the anti-tumor response elicited by MSC-TPr, we next validated efferocytosis of injected cells directly *in vivo*. Hence, an experiment was designed in which CellTrace™-labelled MSC-TPr were injected into immunocompetent syngeneic mice undergoing control liposome or clodronate injection followed by a peritoneal lavage (**Figure 6A**). Analysis of collected cells 4 h later confirmed the absence of myeloid cells in clodronate-treated animals but revealed the presence of two distinct CD11b^+^ cell populations in control liposome-treated mice (**Figure 6B**) with only CD11b^hi^ cells staining positive for CellTrace™ (**Figure 6C**). This salient observation led us to formulate the hypothesis that the therapeutic efficiency of MSC-TPr relies on a balance between efferocytosis and cross-priming of professional antigen-presenting cells. Indeed, OVA-pulsed BM-derived DCs successfully activated CD8 T cells derived from MSC-TPr-vaccinated animals, which was further enhanced if clodronate-treated donor mice are used (**Figure 6D**). Given that phosphatidylserine (PIS) can serve as an “eat-me” signal to monocytes/macrophages, we next quantified the levels of PIS on the surface of live versus heat-killed MSC-TPr (21). The basal level of PIS on live MSC-TPr was substantially higher than that on Ctl MSCs and further enhanced after heat-killing (**Figure 6E**) along with a loss in H2-K^b^ detection on the cell surface (**Figure 6F**). These observations served as impetus to compare the anti-tumor ability of heat-killed MSC-TPr (dead cells with high PIS cell surface levels but no chemokines) versus live MSC-TPr delivered to mice pre-treated with anti-CD11c antibodies as a means to functionally impair DC cross-priming (22). Both treatments impaired the anti-tumor efficacy of the vaccine (**Figure 6G**) clearly indicating that the fate of the immune response induced by MSC-TPr results from a delicate balance between efferocytosis mediated by a subset of CD11^hi^ phagocytes known for mediating immune-suppression, and DC cross-priming responsible for eliciting a pro-inflammatory response.

## Discussion

The discovery of a unique and conserved TPr complex despite constant evolution of adaptive immunity has offered an altered and well accepted peptide model for intrathymic positive selection of competent CD8 T cells (23, 24). However, the impact of the TPr on the proteome and/or transcriptome to pervasively affect given cellular functions besides providing a unique set of MHCI peptides remains a matter of debate (14, 25). Here, we report for the first time the therapeutic use of the TPr complex to mount protective anti-tumor responses using both syngeneic and allogeneic MSCs. Although TPr expression in MSCs did not alter their original mesenchymal phenotype, a wide landscape of transcriptional changes affecting various biological functions including endogenous antigen presentation was observed. We further highlight key roles for host-derived phagocytes and professional antigen-presenting cells in orchestrating the balance for MSC-TPr effector functions (**Graphical Abstract**).

Our results are in agreement with the notion that the TPr complex provides a unique peptide repertoire exhibiting low affinity to the CD8 T-cell receptor (26). This is best exemplified by the inability of MSC-TPr to activate CD8 T cells derived from transgenic or vaccinated animals (26). The impaired activation capacity of MSC-TPr could not be due to an absent OVA-derived peptide repertoire as OVA pulsing enhanced MHCI levels on the surface of MSC-TPr indicating *de novo* peptide-MHCI complex formation. If we presume that the peptide repertoire generated by MSC-TPr is mainly composed of weak agonists or antagonist peptides, then DC-based activation of CD8 T cells derived from MSC-TPr-vaccinated animals shed light on a “bystander” role for MSC-TPr instead of their direct involvement in antigen presentation *in situ.*

Clearance of dying cells by phagocytes (efferocytosis) have recently sparked significant interest in the controversial field of *in vivo*-mediated effector functions of MSCs (20). This concept stipulates the need for the induction of MSC apoptosis *in vivo* in an MHC/HLA-independent fashion but requiring the paracrine effect of granules released by activated cytotoxic cells (20). This concept of inflammation-induced MSC-TPr apoptosis is inconsistent with our syngeneic model as we administered MHC-matched MSCs to naïve animals with no apparent signs of inflammation. An alternative explanation may, in part, involve the higher PIS content on the surface of MSC-TPr, which can serve as an “eat-me” flag signal especially if combined with enhanced recruitment of myeloid cells (phagocytes and DCs) *via* specific chemokines such as CCL2, CX3CL1, and CCL9 (13, 21). This is consistent with the improved anti-tumor responses observed in phagocyte-depleted animals and the lack of therapeutic effects using heat-killed MSC-TPr. If phagocytes impair the anti-tumor response of the vaccine and MSC-TPr cannot behave as an antigen-presenting cell *in vivo*, then how can animals develop a protective OVA-specific CD8 T-cell response? The simplest explanation lies in endogenous DC cross-priming as animals undergoing anti-CD11c treatment are incapable of mounting protective anti-tumor responses (22). Besides, the loss of function observed with heat-killed MSC-TPr also indicate the need for metabolically-fit cells secreting specific chemokines and perhaps other soluble growth factor to recruit and cross-prime endogenous DCs.

## Conclusion

Our findings provide clear evidence that TPr-based proteasomal alterations can trigger potent T-cell immunity when used as part of an engineered MSC-based vaccine. By modulating the antigen processing activity and chemokine secretion profile of MSCs, TPr expression coordinates an indirect but efficient pro-inflammatory response exploiting MSCs as a “living vector with a therapeutic payload” potentially delivering antigen fragments to host-derived DCs. Investigating the nature and exact involvement of the “eat-me” signals as well as the role played by individual chemokines would further define the molecular landscape of this interaction.

## Data Availability Statement

The datasets presented in this study are available online, under repository: GEO accession GSE161773.

## Ethics Statement

The animal studies including all experimental procedures and protocols were reviewed and approved by the Animal Ethics Committee of Université de Montréal.

## Author Contributions

J-PB designed most of the study, carried-out most of the experiments, analyzed the data and prepared the figures. NE-H conducted the bioinformatics analysis for the RNA-Seq. AE-K, JA, NS and RS designed some experiments and wrote some sections of the manuscript. MR designed the study, discussed the results with all authors and wrote the manuscript. All authors contributed to the article and approved the submitted version.

## Funding

This work was supported by a Discovery Grant from the National Sciences and Engineering Research Council of Canada (RGPIN/06101-2014).

## Conflict of Interest

RS is the founder of IntelliStem Technologies Inc. (Toronto, ON, Canada).

The remaining authors declare that the research was conducted in the absence of any commercial or financial relationships that could be construed as a potential conflict of interest.
