# Supplementary material for: Thymoproteasome-Expressing Mesenchymal Stromal Cells Confer Protective Anti-Tumor Immunity via Cross-Priming of Endogenous Dendritic Cells
Source: Front Immunol. 2021 Jan 19;11:596303. doi: 10.3389/fimmu.2020.596303 (PMC7853649; doi:10.3389/fimmu.2020.596303)
Supplement: Supplementary file 1 [file DataSheet_1.pdf]

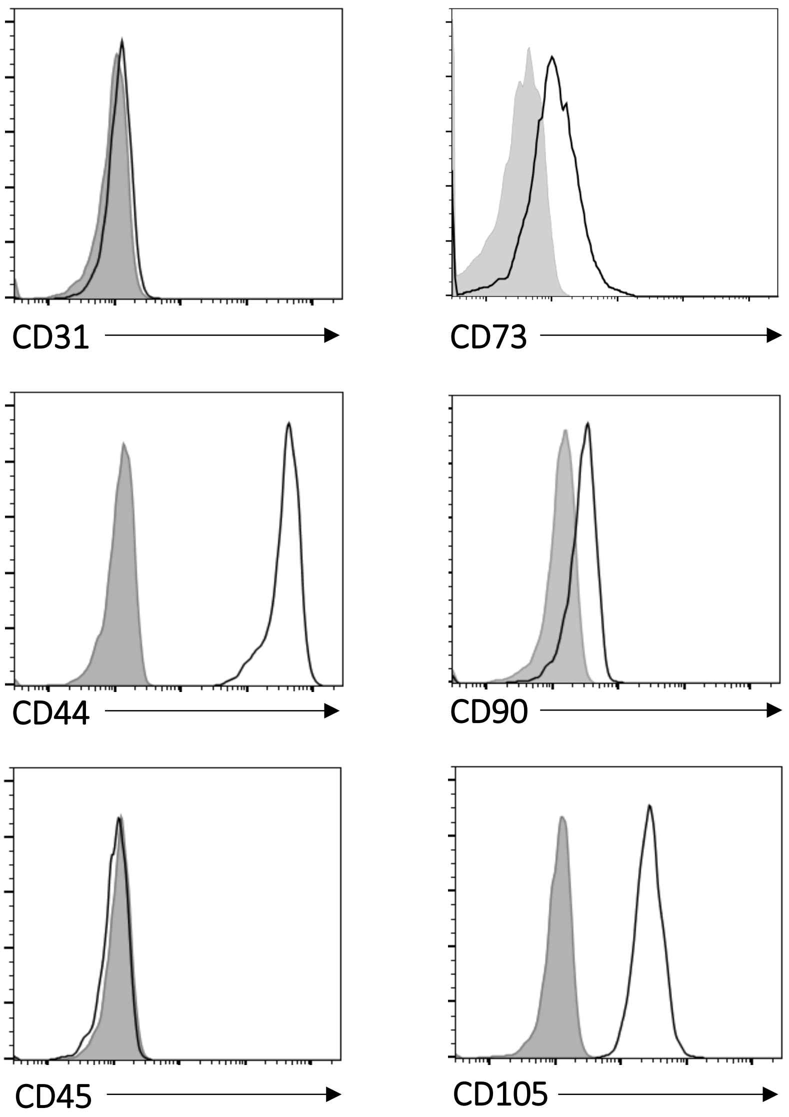

Figure S1: Phenotypic assessment of Ctl MSCs.

Ctl MSCs are negative for the expression of CD31, and CD45 while expressing CD44, CD73, CD90 and CD105. Isotype controls are shown by filled histograms.
