# Supplementary material for: Thymoproteasome-Expressing Mesenchymal Stromal Cells Confer Protective Anti-Tumor Immunity via Cross-Priming of Endogenous Dendritic Cells
Source: Front Immunol. 2021 Jan 19;11:596303. doi: 10.3389/fimmu.2020.596303 (PMC7853649; doi:10.3389/fimmu.2020.596303)
Supplement: Supplementary file 2 [file DataSheet_2.pdf]

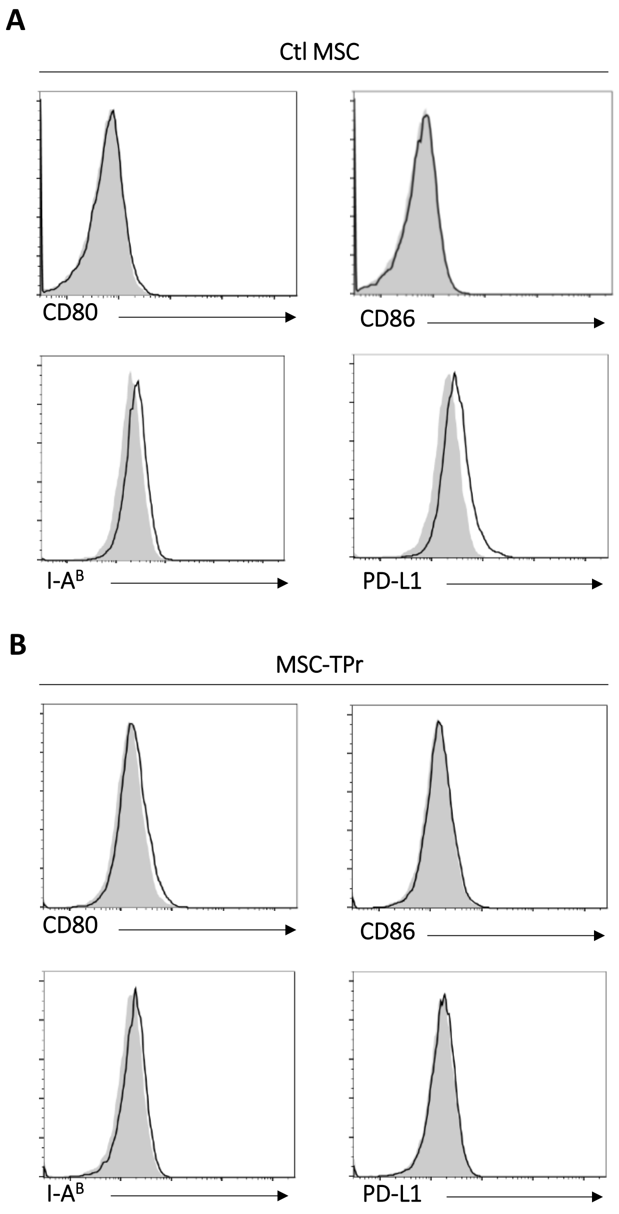

Figure S2: Comparative phenotypic assessment between Ctl and MSC-TPr.

Both Ctl (A) and TPr-expressing MSCs (B) are negative for the expression of CD80, CD86, MHCII (I-A^b) and PD-L1. Isotype control is shown by filled histograms.
