# Supplementary material for: Thymoproteasome-Expressing Mesenchymal Stromal Cells Confer Protective Anti-Tumor Immunity via Cross-Priming of Endogenous Dendritic Cells
Source: Front Immunol. 2021 Jan 19;11:596303. doi: 10.3389/fimmu.2020.596303 (PMC7853649; doi:10.3389/fimmu.2020.596303)
Supplement: Supplementary file 3 [file DataSheet_3.pdf]

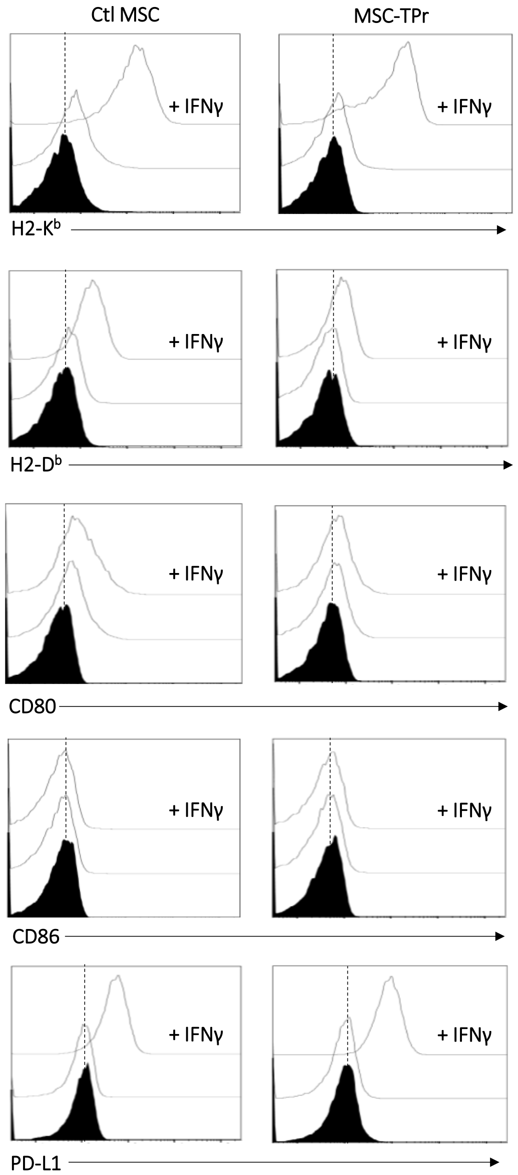

Figure S3: Comparative phenotypic analysis in response to IFN-gamma stimulation.

In response to IFN-gamma treatment (20 ng/ml overnight), both Ctl and MSC-TPr up-regulate the expression of MHCI (H2-K^b and H2-D^b) and PD-L1. However, the expression of both co-stimulatory molecules (CD80 and CD86) remain negative. Isotype control is shown by black histograms.
