# Supplementary material for: Thymoproteasome-Expressing Mesenchymal Stromal Cells Confer Protective Anti-Tumor Immunity via Cross-Priming of Endogenous Dendritic Cells
Source: Front Immunol. 2021 Jan 19;11:596303. doi: 10.3389/fimmu.2020.596303 (PMC7853649; doi:10.3389/fimmu.2020.596303)
Supplement: Supplementary file 4 [file DataSheet_4.pdf]

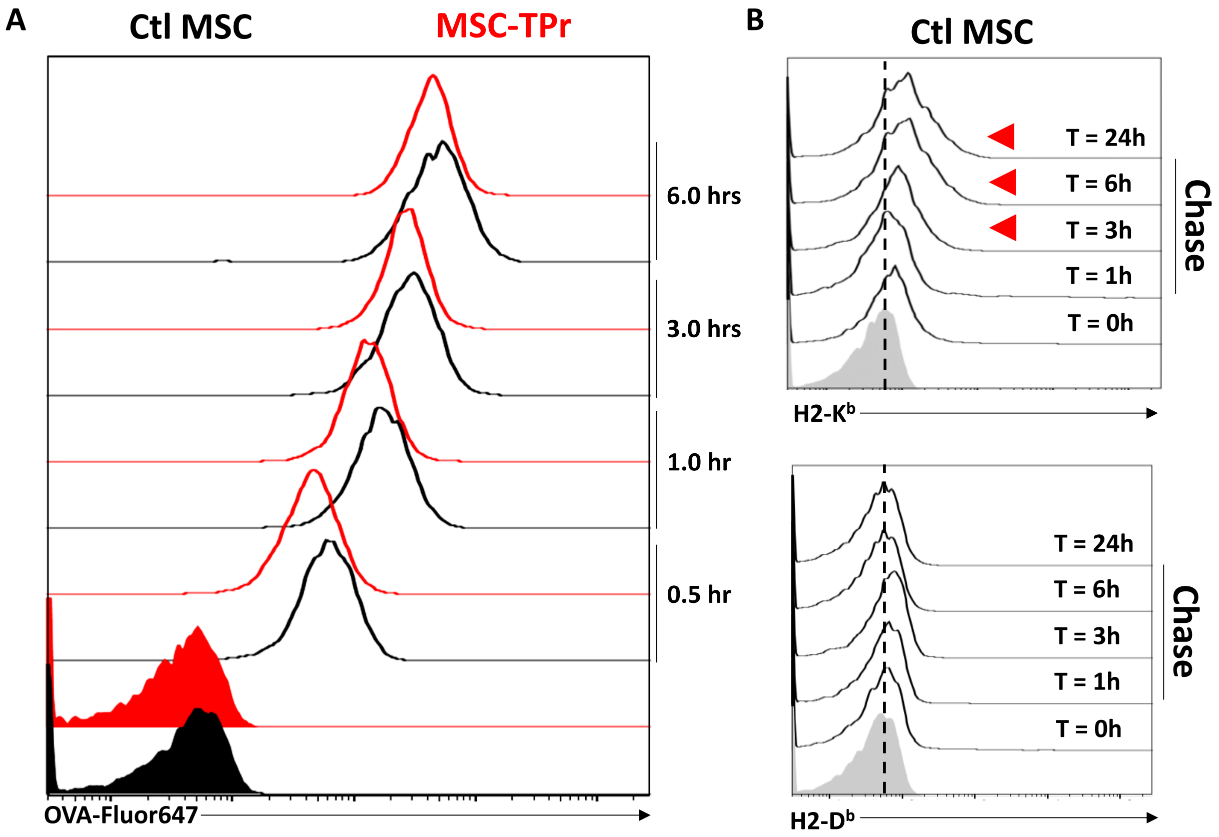

Figure S4. Comparative analysis of OVA up-take and MHCI upregulation on Ctl MSCs in response to OVA pulsing.

A) Representative flow-cytometry assessment of fluorescent OVA uptake by Ctl MSCs (black) and MSC-TPr (red) at different time points. Both cell populations were treated with 1µg/ml of OVA-647. **B)** Cell surface expression of MHCI molecules was assessed by flow-cytometry in response to OVA pulsing for 1h (5 mg/ml). After washing, cells were analyzed at the depicted time points. Grey histograms represent non-pulsed cells.
